# Evaluation of Antioxidant and DNA Damage Protection Activity of the Hydroalcoholic Extract of *Desmostachya bipinnata* L. Stapf

**DOI:** 10.1155/2014/215084

**Published:** 2014-01-19

**Authors:** Upendarrao Golla, Solomon Sunder Raj Bhimathati

**Affiliations:** ^1^Department of Biological Sciences, Indian Institute of Science Education and Research (IISER) Bhopal, Bhopal, Madhya Pradesh 462023, India; ^2^Department of Pharmacology, Faculty of Pharmaceutical Sciences, Bharat Institute of Technology, Hyderabad, Andhra Pradesh 501510, India

## Abstract

*Desmostachya bipinnata* Stapf (Poaceae/Gramineae) is an official drug of ayurvedic pharmacopoeia. Various parts of this plant were used extensively in traditional and folklore medicine to cure various human ailments. The present study was aimed to evaluate the antioxidant and DNA damage protection activity of hydroalcoholic extract of *Desmostachya bipinnata* both *in vitro* and *in vivo*, to provide scientific basis for traditional usage of this plant. The extract showed significant antioxidant activity in a dose-dependent manner with an IC_50_ value of 264.18 ± 3.47 **μ**g/mL in H_2_O_2_ scavenging assay and prevented the oxidative damage to DNA in presence of DNA damaging agent (Fenton's reagent) at a concentration of 50 **μ**g/mL. Also, the presence of extract protected yeast cells in a dose-dependent manner against DNA damaging agent (Hydroxyurea) in spot assay. Moreover, the presence of extract exhibited significant antioxidant activity *in vivo* by protecting yeast cells against oxidative stressing agent (H_2_O_2_). Altogether, the results of current study revealed that *Desmostachya bipinnata* is a potential source of antioxidants and lends pharmacological credence to the ethnomedical use of this plant in traditional system of medicine, justifying its therapeutic application for free-radical-induced diseases.

## 1. Introduction

Complementary systems of medicine include, namely, Siddha, Ayurveda, Kaempo, and Unani, and Chinese medicines have gained their attractiveness in recent years [1]. The demand for herbal medicines is greater than ever due to their safety, efficacy, less side effects, and good belief of society in herbal medicines and their products [2]. Medicinal plants are significant source of synthetic and herbal drugs have been used for the treatment or prevention of diseases and for the promotion of good health since antiquity. Many of the drug molecules in modern pharmacology are derived from plant sources [3]. Plants' secondary metabolites are tremendous resource to develop new drugs and exhibit numerous biological activities like antifungal, anticancer, and antibacterial and antioxidants that are utilized in food, agricultural, and pharmaceutical industries [4, 5]. Because of the probable toxic effects of synthetic antioxidants like BHA (butylated hydroxyl anisole) and BHT (butylated hydroxyl toluene) and natural antioxidants especially from plant gained major attention and importance towards treatment of various free-radical-related diseases such as cancer, asthma, atherosclerosis, arthritis, aging, and autoimmune disorders, several stress-related diseases including cataracts, cognitive dysfunction, myocardial infarction, and diabetes, and several cardiovascular and neurodegenerative diseases [6, 7]. The intake of synthetic and natural antioxidant products has been shown to reconcile their effect mainly due to redox properties which allows them to act as hydrogen donators, reducing agents, and singlet oxygen quenchers [8].

In continuation of our efforts to corroborate the efficiency of traditional medicine, we have selected *Desmostachya bipinnata* based on the ethnopharmacological information. *Desmostachya bipinnata *L. Stapf (Poaceae/Gramineae) is an official drug of ayurvedic pharmacopoeia, commonly called sacrificial grass [9]. It is distributed throughout India, also found in Egypt, Nubia, and Syria, Persia, Pakistan, Middle East to Indo-China, and North and tropical Africa. In Indian traditional and folkloric medicine, various parts of this plant were extensively used to cure diarrhea, dysentery, jaundice, dysuria, vomiting, menorrhagia, skin eruptions, urinary calculi, and other diseases of skin and bladder. In addition, these plant parts were used as a galactogogue, astringent, aphrodisiac, antiinflammatory, antiasthmatic, antipyretic, analgesic, diuretic, and sedative to pregnant uterus [10, 11].

Previous studies on this plant resulted in the isolation of some known coumarins (scopoletin and umbelliferone), amino acids, carbohydrates [12], flavonoids like kaempferol, quercetin, quercetin-3-glucoside, trycin and trycin-7-glucoside [13, 14], sterols [15], and terpenes [16]. Pharmacological studies on *Desmostachya bipinnata *established its antiulcerogenic [13], analgesic, antipyretic, and anti-inflammatory activities [16, 17], antidiarrhoeal activity [18], antifungal [19, 20] and antihelicobacter activities [14], and antimicrobial activity and antibacterial activity against* Staphylococcus aureus, Escherichia coli*, and *Klebsiella pneumoniae *[21]. A new xanthene (2,6-dihydroxy-7-methoxy-3H-xanthen-3-one) was isolated from the methanolic extract of *Desmostachya bipinnata*, which was found to inhibit signal transducer and activator of transcription 3 (STAT3) and low-density lipoprotein-oxidation [22]. Interestingly, feeding with *Desmostachya bipinnata* had been shown to prolong nymphal development for 2nd, 5th, and 6th instars of Grasshopper *Hieroglyphus nigrorepletus* [23] and failed to show lethality or cytotoxicity on brine shrimps which supports its safe use for human consumption [24].

Considering the pharmacological importance of this plant, it is necessary to investigate its antioxidant and free radical scavenging properties as there is emergent role of free radicals in disease progression. Therefore, this study was aimed at evaluation of antioxidant and DNA damage protection properties of hydroalcoholic extract of this plant *in vitro* and *in vivo* using suitable models to provide scientific basis, to justify its folkloric usage.

## 2. Materials and Methods

### 2.1. Plant Material

The plant *Desmostachya bipinnata *L. Stapf was collected in and around Nalgonda city, Andhra Pradesh, India. The plant material was botanically authenticated by Dr. T. Shankara Chary, Government Degree College for Women, Nalgonda, India. The voucher specimen (number DBP/GDCWN/54/2010) was deposited in the college herbarium for future reference.

### 2.2. Preparation of Extract

The whole plant of *Desmostachya bipinnata* was shade-dried, powered coarsely (sieve number 40), and then extracted in a Soxhlet extractor using 70% of methanol as a solvent at 55°C until the extractive becomes colorless. The filtrate obtained by vacuum filtration was concentrated to dryness using vacuum evaporator under controlled temperature (40–50°C) [24]. The dried concentrated extract was suspended in water for study.

### 2.3. Chemicals and Reagents

L-Ascorbic acid, agarose, and hydroxyurea (HU) were purchased from Sigma Aldrich, India. The yeast growth media components and hydrogen Peroxide were purchased from Merck, India. Ferric chloride was purchased from Himedia, India. All other reagents and chemicals used in this work were of analytical grade and obtained commercially from the regular store suppliers.

### 2.4. Yeast Strain, Media, and Growth Conditions

The *Saccharomyces cerevisiae *wild-type strain BY4741 (MAT*α* his3Δ1 leu2Δ0 met15Δ0 ura3Δ0) was provided by Peter Svensson & Samson Lab (Department of Biological Engineering, Massachusetts Institute of Technology, Cambridge, MA, USA). Cells were grown up to the middle of first exponential phase (10^6^ cells/mL), OD_600_ between 0.6 and 1 in liquid YPD medium (1% yeast extract, 2% glucose, and 2% peptone) using an orbital shaker at 28°C and 160 rpm. Solid Hydroxyurea (150 mM) laden YPD media plates were prepared by adding filter-sterilized Hydroxyurea stock solution to the autoclaved YPDA media (1% yeast extract, 2% glucose, 2% peptone, and 2% agar).

### 2.5. Preliminary Phytochemical Screening

The crude hydro-alcoholic extract of *Desmostachya bipinnata* was subjected to preliminary qualitative phytochemical screening for the identification of major functional groups and various phytochemical constituents such as carbohydrates, glycosides, alkaloids, flavonoids, saponins, tannins, phenolic compounds, terpenoids, steroids, proteins, gums, and mucilage using standard tests [25, 26].

### 2.6. *In Vitro* Antioxidant Activity

#### 2.6.1. Hydrogen Peroxide (H_2_O_2_) Radical Scavenging Assay

The ability of hydroalcoholic extract of* Desmostachya bipinnata* to reduce hydrogen peroxide was assessed by the method described by Gülçin et al. [27]. A solution of 40 mM hydrogen peroxide was prepared in phosphate buffer (pH 7.4). Both the plant extract and Ascorbic acid were dissolved in distilled water and 1 mL of test extract (or) Ascorbic acid in different concentrations (50, 100, 200, 300, 400, and 500 *μ*g/mL) was added to a 0.6 mL of 40 mM hydrogen peroxide solution. The mixture was allowed to incubate at room temperature for 30 minutes. The absorbance of hydrogen peroxide was determined at 230 nm using UV Spectrophotometer (Shimadzu, UV 1601) against corresponding blank solution containing phosphate buffer solution without hydrogen peroxide to avoid background. The absorbance of hydrogen peroxide in phosphate buffer was used as control and Ascorbic acid as positive control. The experiment was performed in triplicate. The percentage scavenging of hydrogen peroxide of samples and Ascorbic acid were calculated using the following formula:

(1) $$\begin{array}{c} \%{\text{H}}_{2}{\text{O}}_{2}\;\text{scavenging activity}=\left[\frac{A_{0}-A_{I}}{A_{0}}\right]\times 100, \end{array}$$

where *A*_0_ is the absorbance of the control and *A*_*I*_ is the absorbance of the plant extract.

#### 2.6.2. DNA Protection Assay

The ability of different concentrations of plant extract to protect pUC19 plasmid DNA from harmful effects of hydroxyl radicals produced by Fenton's reagent was evaluated by DNA nicking assay as described earlier [28] with minor modifications. The reaction mixture contained 3 *μ*L of plasmid DNA, 10 *μ*L of Fenton's reagent (30 mM H_2_O_2_, 50 mM Ascorbic acid, and 80 mM FeCl_3_) followed by the addition of different concentrations of extract (0, 5, 10, 15, 30, and 50 *μ*g/mL) and the final volume of the mixture was brought up to 20 *μ*L using double-distilled water. The reaction mixtures were allowed to incubate for 30 min at 37°C. After 30 min incubation, bromophenol blue dye (0.25% in 50% glycerol) was added. The reaction mixtures (20 *μ*L) were loaded on 0.8% agarose gel (prepared by dissolving 0.4 g of agarose in 50 mL of 1 × TBE Buffer) and electrophoresis was carried out at 90 V for 1 hour followed by ethidium bromide staining. The closed circular, linear, and relaxed forms of pUC19 were visualized and quantified using LAS-4000 MINI Gel Documentation system.

#### 2.6.3. Spot Assay

Wild-type *Saccharomyces cerevisiae* (BY4741 strain) was used to investigate the effect of extract on the growth of yeast cells, to select the doses of extract used in the adaptive treatments. *Saccharomyces cerevisiae* cells (OD_600_ of 0.6–1) without and with exposure to increased concentrations of extract (200, 400, and 800 *μ*g/mL) and stressing agent (2 mM) were incubated in a YPD medium for 1 hr at 28°C/160 rpm. In all cases, about 3 *μ*L of yeast saturated cultures after 1 hr incubation was serially diluted (10^−1^, 10^−2^, 10^−3^, and 10^−4^) and was dropped on standard YPDA plates and Hydroxyurea (150 mM) laden YPDA plates. Plates were incubated at 28°C, and growth was recorded after 24, 48, and 72 hrs by scanning the plates using HP scanner. The lowest concentration was chosen which could improve cell growth compared to cohorts exposed to stress without being treated with plant extract.

### 2.7. *In Vivo* Antioxidant Activity

*In vivo* antioxidant activity was carried out using eukaryotic cells of the yeast as already described [29, 30]. *Saccharomyces cerevisiae* (wild-type strain BY4741) treated without and with the highest noncytotoxic concentrations 200, 400, and 800 *μ*g/mL of extract along with oxidative stressing agent H_2_O_2_ (4 mM). Cells at exponential phase were treated with extract for 1 hr at 28°C/160 rpm before being stressed. Then, cells were stressed with hydrogen peroxide (4 mM) by incubating for 2 hr at 28°C/160 rpm. In all cases, after incubation, samples were seeded or spread in triplicate on YPDA plates after proper dilution in sterilized liquid YPD medium. Plates were incubated for 48 hr at 28°C; later on colonies were counted and compared to the control plates (untreated cells), which were considered to represent 100% survival of the yeast cell. The antioxidant activity of the extracts was evaluated by the ability of the extracts to prevent (or) minimize the oxidative lethal damages induced by H_2_O_2_. Tolerance was expressed as percentage of survival [31].

## 3. Results and Discussion

### 3.1. Preliminary Phytochemical Screening

The whole plant of *Desmostachya bipinnata *was extracted using methanol (70%) as solvent in Soxhlet extractor, which has given reddish brown sticky mass. The percentage yield of hydroalcoholic extract was found to be 5.4% w/w.

The preliminary phytochemical screening of crude hydroalcoholic extract revealed the presence of phytochemical constituents such as alkaloids, carbohydrates, proteins, tannins, phenolic compounds, flavonoids, terpenoids, and glycosides whereas steroids, saponins, and gums and mucilage were absent. The results were shown in Table 1.

### 3.2. *In Vitro* Antioxidant Activity

#### 3.2.1. Hydrogen Peroxide (H_2_O_2_) Radical Scavenging Assay

Hydrogen peroxide is a weak oxidizing agent that inactivates a few enzymes directly, usually by oxidation of essential thiol (–SH) groups. It can cross cell membranes rapidly; once being inside the cell, it can react with Fe^2+^ and Cu^2+^ ions to form hydroxyl radicals and this may be the origin of many of its toxic effects [32]. Thus, removing H_2_O_2_ as well as O^2−^ is very important for protection of food systems.

**Figure 1:**
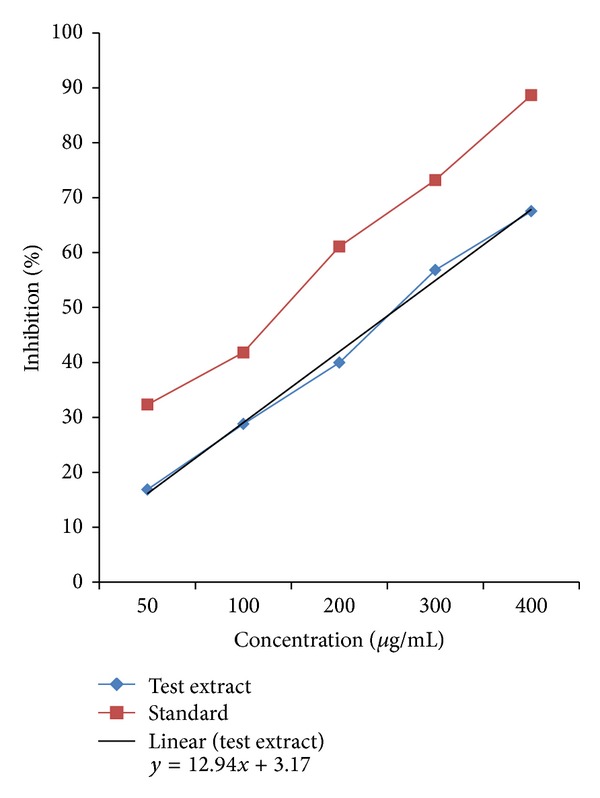
H_2_O_2_ radical scavenging assay for hydro-alcoholic extract of *Desmostachya bipinnata.*

The scavenging ability of the hydro-alcoholic extract of *Desmostachya bipinnata* (DBPS) at different concentrations (50, 100, 200, 300, and 400 *μ*g/mL) on hydrogen peroxide was expressed as % inhibition and compared with that of standard Ascorbic acid. The result was shown in Figure 1. The IC_50_ value for the DBPS extract and standard Ascorbic acid was 264.18 ± 3.47 and 147.63 ± 5.25 *μ*g/mL, respectively. From the result, it was clear that the *Desmostachya bipinnata *(DBPS) extract exhibited significant H_2_O_2_ radical scavenging activity in a dose-dependent manner when compared to that of the standard ascorbic acid.

The antioxidant components present in the extract are good electron donors, which may accelerate the reduction of H_2_O_2_ to H_2_O. Dietary polyphenols, especially compounds with the orthodihydroxy phenolic structure catechin, quercetin, gallic acid ester, and caffeic acid ester, have been shown to protect mammalian and bacterial cells against hydrogen-peroxide-induced cytotoxicity [33, 34]. Therefore, the phenolic compounds of the hydro-alcoholic extract of *Desmostachya bipinnata* or other flavonoids present in extract may be responsible for its H_2_O_2_ radical scavenging or antioxidant activity.

#### 3.2.2. DNA Protection Assay

This assay was based on the ability of extracts to protect the pUC19 plasmid DNA against damage caused by hydroxyl (^•^OH) radicals. Hydroxyl radicals generated by the Fenton reaction are known to cause oxidatively induced breaks in DNA strands to yield its open circular or relaxed forms. Exposure of plasmid DNA to Fenton's reagent ultimately results in strand breaks, mainly due to the generation of reactive species-hydroxyl radical and the subsequent free radical-induced reaction on plasmid DNA. Hydroxyl radicals react with nitrogenous bases of DNA producing base radicals and sugar radicals. The base radicals in turn react with the sugar moiety causing breakage of sugar phosphate backbone of nucleic acid, resulting in strand break [35].

**Figure 2:**
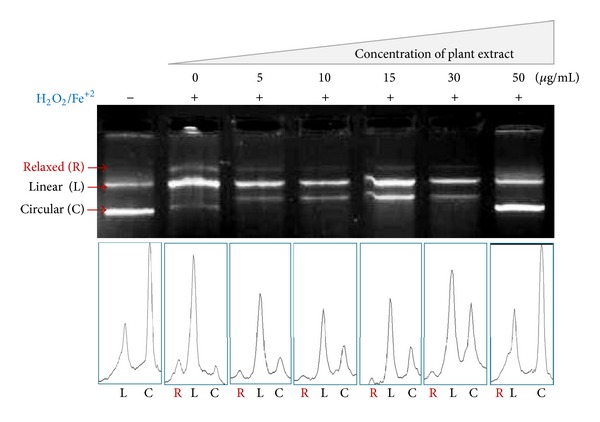
DNA protection assay of *Desmostachya bipinnata* hydroalcoholic extract. Treatment of pUC19 plasmid with hydroalcoholic extract in absence and presence of DNA nicking/damage causing Fenton's reagent followed by the densitometric analysis of relaxed (R), linear (L), and circular (C) forms of pUC19 DNA (underneath agarose gel).

The scavenging effect of extract was further evaluated in plasmid nicking assay.  Figure 2 depicts the ability of the extracts to reduce Fe^+3^-dependent plasmid DNA nicking. When plasmid DNA was dissolved in Fenton's reagent, it resulted in the formation of single-stranded relaxed nicked DNA (R-Form) and double-stranded nicked and linear DNA (L-Form). Addition of extract resulted in the formation of native circular (C) plasmid DNA formation, causing disappearance of Fe^+3^-mediated linear (L) and relaxed (R) forms of plasmid DNA. The quantification of three forms of plasmid DNA was carried out using ImageJ software and shown underneath the agarose gel. As shown in Figure 2, the circular form (C) of untreated DNA was converted into nicked forms like relaxed (R) and linear (L) DNA, and upon treatment with extract regained its native form of DNA into circular form (C) and protected DNA from the hydroxyl (^•^OH) radical (i.e., Fenton's reagent) induced oxidative DNA damage.

**Figure 3:**
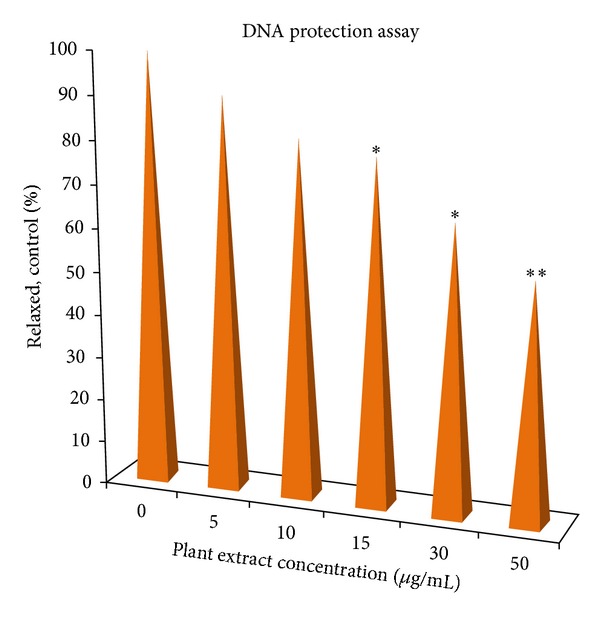
The percentage protection of pUC19 DNA by the extract. Values are expressed as mean of triplicate measurements; *P < 0.05, **P < 0.01.

Further densitometric analysis was carried out using ImageJ software and the quantification of relaxed form of DNA (R-Form) relative to the untreated reaction. The quantification of relaxed form of DNA, shown in Table 2, revealed that the extract was effective in protecting DNA by inhibiting the nicking caused by Fenton's reagent as shown in Figure 3. So, hydro-alcoholic extract of *Desmostachya bipinnata* (DBPS) showed significant reduction in the formation of nicked DNA and increased native form of plasmid DNA at the concentration of 50 *μ*g/mL. The protective effect of extract on DNA may be attributed to the presence of flavonoids and phenolic compounds like trycin and quercetin which can prevent the production of ROS by complexing cations such as copper and iron that participate in hydroxyl radical formation [36].

#### 3.2.3. Spot Assay

**Figure 4:**
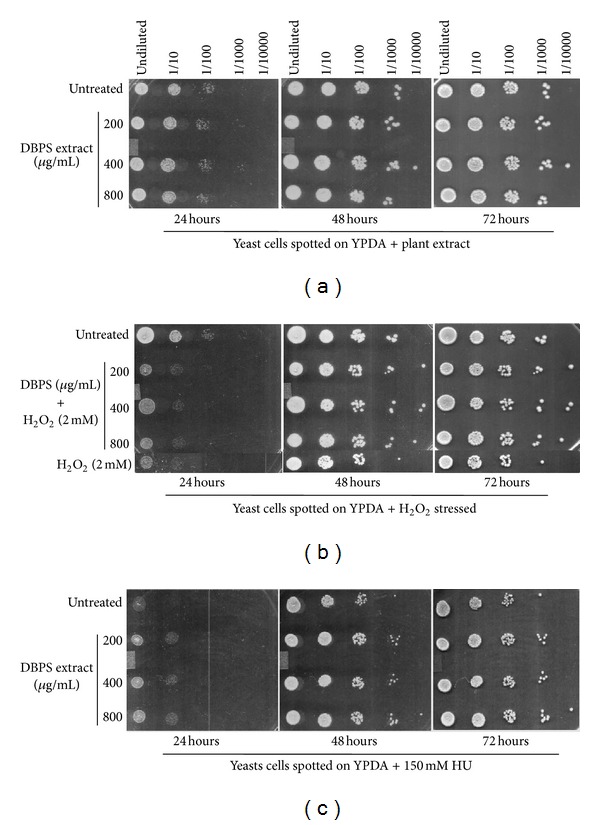
(a) Growth of extract treated yeast cells in comparison to untreated cells. (b) Growth of extract treated yeast cells in presence of stressing agent (H_2_O_2_) in comparison to untreated cells. (c) Growth of yeast cells on Hydroxyurea (HU) laden YPDA plates with and without treatment of extract.

The growth of yeast cells untreated and treated with DBPS extract in three different concentrations was shown in Figure 4(a). There was no toxicity of extract observed at tested concentrations. So, these concentrations of plant extract were used for further studies. The growth test of yeast cells stressed with H_2_O_2_ (2 mM) in presence and absence of DBPS extract had shown significant growth difference and extract treated cells growing normally in presence of stressing agent in comparison to growth of unstressed control cells as shown in Figure 4(b). This shows that the protective effect of DBPS extract may be attributed to the presence of various reported antioxidant compounds.

In addition, the growth test of yeast cells on Hydroxyurea (150 mM) laden YPDA plates in presence and absence of DBPS extract had shown significant growth difference. The extract treated cells grow normally in presence of DNA damaging agent (Hydroxyurea) in comparison to growth of untreated control cells as shown in Figure 4(c).

Hydroxyurea (HU) is a known chemotherapeutic agent, commonly used for the treatment of solid tumors and myeloproliferative disorders (MPD). Because of carcinogenic and mutagenic potential of HU, it is commonly used in both prokaryotes (*Escherichia coli*) and eukaryotes (yeast) to study DNA-damage-dependent processes. Hydroxyurea induces the formation of site-specific DNA damage through formation of hydrogen peroxide and nitric oxide [37, 38]. The protection of yeast cells by DBPS extract on HU laden YPDA plates was in accordance with the above observations and suggests the probable role of extract in scavenging hydrogen peroxide (H_2_O_2_) radical. This indicates the role of extract in protection of cells against DNA damaging agents and substantiates its antioxidant activity.

### 3.3. *In Vivo* Antioxidant Activity

**Figure 5:**
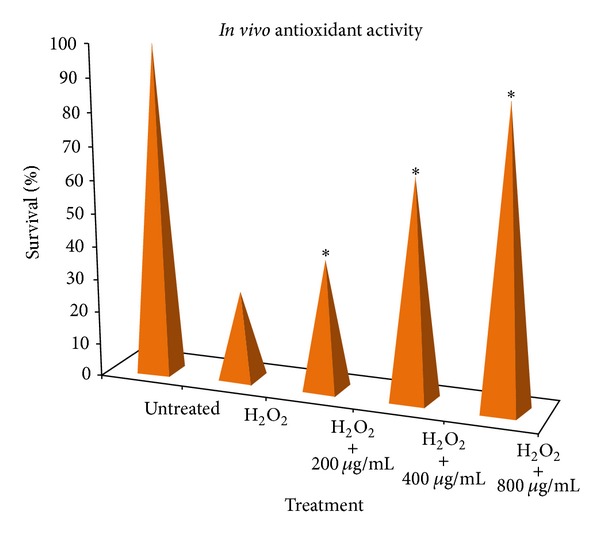
Survival of *S. cerevisiae* cells untreated and treated with DBPS extract in presence of stressing agent hydrogen peroxide (4 mM). *Significantly different from the stressing agents by the analysis of variance (ANOVA) and Dunnett's *t*-test (*P* ≤ 0.05).

The hydro-alcoholic extract of DBPS had shown significant antioxidant activity *in vivo* by increasing the %survival of yeast cells against a stressing agent (H_2_O_2_). The results were shown in Table 3 and graphically represented in Figure 5. The %survival of yeast cells was measured in triplicate and was based on the number of colonies observed after 24 hr incubation of extract treated and untreated yeast cells in presence of stressing agent in comparison to only stressed cells. The present investigation on *Desmostachya bipinnata *hydroalcoholic extract for antioxidant activity *in vitro* and *in vivo* has shown significant results and its antioxidant; DNA protection activity may be attributed to the presence of various antioxidant phytochemical constituents such as polyphenolic compounds, flavonoids, and phenolic acids which were reported to neutralize different free radical agents like superoxide, hydroxyl (OH), hydrogen peroxide (H_2_O_2_), and peroxyl (ROO) by diverse mechanisms including electron donation as reducing agent and metal chelation [39].

Free radicals called reactive oxygen species (ROS) are normal products of human metabolism. The human body evolved a mechanism to neutralize the oxidative stress by producing antioxidants naturally *in situ* such as catalase, superoxide dismutase, and glutathione peroxidase. These antioxidant enzymes may be supplied externally through foods and supplements [40]. However, antioxidant defense mechanism does not meet demand in the case of increase of ROS level in several diseases. Damage on the tissues occurs by isoprostanes generated by lipid peroxidation, increase in the content of aldehydes, or protein carbonyls produced from protein oxidation and oxidized base adducts generated from DNA oxidation [41]. Nowadays, a large number of studies reported antioxidant activities for plant-based substances, thus resulting in evolution of herbal drugs in complementary and alternative folkloric medicine in the world.

## 4. Conclusion

Preliminary phytochemical analysis revealed that the hydro-alcoholic extract of DBPS contains alkaloids, carbohydrates, proteins, tannins, phenolic compounds, flavonoids, terpenoids, and glycosides. In addition, the DBPS extract exhibited significant free radical scavenging and antioxidant activity against hydrogen peroxide radicals both *in vitro* and *in vivo*. Moreover, the DBPS extract showed significant protection activity against Fenton's reagent and HU-induced DNA damage on pUC19 DNA and yeast cells, respectively. These activities of DBPS extract may be attributed to the presence of various phytochemical constituents such as polyphenolics, flavonoids, and phenolic acids which were reported earlier to neutralize different free radicals. Although a large number of active compounds are isolated from DBPS, further studies are needed for isolation, structural elucidation, and screening of any of the above-mentioned active principles to proposed activity of drug. This study provides experimental evidence and supports the folkloric use of this plant and lends pharmacological credence to the ethnomedical use in traditional system of medicine. Also, this study demands further studies to elaborate its use, active constituents, and safety.

## Figures and Tables

**Table 1:** Preliminary phytochemical screening of hydroalcoholic extract of *Desmostachya bipinnata*.

S. no.	Phytochemical tests	Inference
1	Alkaloids	+
2	Carbohydrates	+
3	Proteins	+
4	Tannin's and phenolic compounds	+
5	Flavonoids	+
6	Steroids	−
7	Triterpenoids	+
8	Saponins	−
9	Glycosides	+
10	Gums and mucilage	−

“+” indicates the presence and “−” indicates the absence of that phytochemical constituent.

**Table 2:** Inhibition of DNA nicking by hydroalcoholic extract of *Desmostachya bipinnata *against Fenton's reagent.

DBPS Extract concentration (*μ*g/mL)	Quantification of relaxed form (R) of DNA	Relative %control
0	109.274 ± 0.82	100 ± 1.32
5	99.187 ± 1.28	90.76 ± 2.16
10	90.24 ± 2.26	82.58 ± 1.72
15	87.23 ± 1.42	79.82 ± 0.94*
30	72.136 ± 1.71	66.01 ± 1.26*
50	59.854 ± 1.17	54.77 ± 2.54**

The relaxed form of DNA quantified relative to control treated with Fenton's reagent but no extract and the values of triplicate measurements are expressed as Mean ± SD; *P < 0.05, **P < 0.01.

**Table 3:** Percentage survival of untreated and extract-treated *S. cerevisiae* cells in presence of oxidative stressing agent (H_2_O_2_).

Treatment	% Survival^a^
Untreated cells (control)	100 ± 1.52
4 mM H_2_O_2_	26.80 ± 0.58^b^
H_2_O_2_ + 200 *μ*g/mL extract	39.17 ± 2.08^c^
H_2_O_2_ + 400 *μ*g/mL extract	65.98 ± 1.15^c^
H_2_O_2_ + 800 *μ*g/mL extract	88.66 ± 0.58^c^

^a^Average ± SD (*n* = 3). ^b^Significantly different from the untreated cells (control) by the analysis of variance (ANOVA) and Dunnett's *t*-test (*P* ≤ 0.05). ^c^Significantly different from the stressing agents by the analysis of variance (ANOVA) and Dunnett's *t*-test (*P* ≤ 0.05).
